# Significance of Neutrophil Gelatinase-Associated Lipocalin (NGAL) and Proprotein Convertase Subtilisin/Kexin Type 9 (PCSK9) for the Monitoring of Treatment Response to Cyclosporine in Patients with Psoriasis

**DOI:** 10.3390/life13091873

**Published:** 2023-09-06

**Authors:** Aleksandra Frątczak, Bartosz Miziołek, Agnieszka Łupicka-Słowik, Marcin Sieńczyk, Karina Polak, Beata Bergler-Czop

**Affiliations:** 1 Department of Dermatology, School of Medicine in Katowice, Medical University of Silesia, 20/24 Francuska St., 40-067 Katowice, Poland; ola.fratczak89@gmail.com (A.F.); m.carrine@gmail.com (K.P.); bettina2@o2.pl (B.B.-C.); 2 Department of Organic and Medicinal Chemistry, Faculty of Chemistry, Wroclaw University of Science and Technology, 50-370 Wroclaw, Poland; agnieszka.lupicka-slowik@pwr.edu.pl (A.Ł.-S.); marcin.sienczyk@pwr.edu.pl (M.S.)

**Keywords:** psoriasis, PCSK9, NGAL, cyclosporine

## Abstract

Neutrophil gelatinase-associated lipocalin (NGAL) may promote development of inflammation in psoriasis, whereas proprotein convertase subtilisin/kexin type 9 (PCSK9) may account for dyslipidemia in some psoriatic patients. The aim of the study was to analyze the influence of cyclosporine therapy on serum levels of NGAL and PCSK9 in patients with psoriasis vulgaris. Methods: Serum samples were obtained before and after three months cyclosporine therapy. Patients were grouped into responders and non-responders to cyclosporine depending on whether they achieved at least 50% reduction of Psoriatic Activity Score Index (PASI), or not. Serum levels of PCSK9 and NGAL were assayed using commercially available ELISA tests. Lipid levels were measured with an enzymatic method. Results: There were 40 patients enrolled. A significant decrease in serum NGAL level was seen in cyclosporine responders. No similar dependance was found for PCSK9. Serum PCSK9 concentration correlated with total cholesterol (TChol) and LDL at baseline and after three month treatment. Conclusions: Cyclosporine therapy contributes to the reduction of the NGAL serum but not the PCSK9 concentration. Correlation between the PCSK9 serum level and TChol as well as LDL concentration may help to understand drug induced dyslipidemia after cyclosporine.

## 1. Introduction

Psoriasis is a chronic inflammatory skin disease which frequently coexists with lipid abnormalities. Mechanisms responsible for the inflammatory response and lipid disturbances in psoriasis remain under ongoing investigations. Neutrophil gelatinase-associated lipocalin (NGAL) was found to participate in the pathogenesis of psoriasis via activation of neutrophils [1] and enhancement of T-helper (Th) 1/Th17-mediated inflammation [2]. Proprotein convertase subtilisin/kexin type 9 (PCSK9) is a molecule which primarily prevents low-density lipoprotein (LDL) receptor from recycling in hepatocytes, therefore promoting an accumulation of circulating LDL [3], but it was also found to promote proliferation and impaired differentiation of keratinocytes [4]. Both NGAL and PCSK9 can be produced by keratinocytes [1,5]. Their tissue expression was shown to be enhanced within the lesioned skin in psoriasis [4,5,6,7] and their serum levels were increased in psoriatic patients [3,8]. A decrease in tissue NGAL expression was previously observed after biologics (risankizumab, ustekinumab) [9] and the narrow-band UVB phototherapy (nb-UVB) [10]. Methotrexate was shown to significantly reduce the level of PCSK9 in serum of psoriatic patients, while acitretin caused its marked increase [11]. The change of NGAL and PCSK9 after cyclosporine therapy has not yet been investigated in psoriasis. The aim of this study was to analyze the influence of cyclosporine treatment on the NGAL and PCSK9 levels in patients with psoriasis vulgaris.

## 2. Materials and Methods

Patients with psoriasis vulgaris were enrolled at the Department of Dermatology between October 2021 and November 2022. Prior to the enrollment they were provided with a written informed consent form, and the study was earlier approved by the local ethical committee at the Medical University of Silesia in Katowice, Poland (PCN/CBN/0022/KB1/46/I/21). Each patient was evaluated towards arterial hypertension and other cardiovascular comorbidities, diabetes mellitus, as well as previous treatment other than topical (acitretin, methotrexate, nb-UVB and psoralen plus UVA therapy). Inclusion criteria involved no systemic treatment of psoriasis as well as immunosuppressive therapies for other reasons within the previous three months. Only topical therapies and nb-UVB phototherapy were allowed soon before the enrollment to the study. Exclusion criteria were other variants of psoriasis (pustular one, isolated nail psoriasis, psoriatic arthritis), undergoing lipid lowering treatments (statins, fibrates), familiar dyslipidemia and infections within the preceding two weeks, insulin-dependent diabetes mellitus, smoking of cigarettes. Skin involvement was evaluated using Psoriatic Activity Score Index (PASI) and Body Surface Area (BSA). Patients were grouped into responders and non-responders to cyclosporine depending on whether they achieved at least 50% reduction of PASI or not. Blood samples were taken from each patient’s antecubital vein after 12–14 h of fasting. The blood samples were collected in a clot activator tube and centrifuged at 1996 RCF for 15 min to obtain serum sample, which was then stored at −80 °C until assayed. The levels of PCSK9 and NGAL in serum were assayed using commercially available ELISA tests obtained from Thermo-Fisher (Waltham, MA, USA). For the PCSK9 assay, the samples were diluted 2-fold, and for the NGAL assay, the samples were diluted 20-fold. Each test was performed in duplicate. The sensitivity of ELISA kit for PCSK9 (#EH384RB) was 1.2 ng/mL with detection range of 1.2–300 ng/mL. The kit for the assay of NGAL (#EHLCN2) had 4 pg/mL sensitivity and 4.1–1000 pg/mL detection range. The levels of C-reactive protein (CRP) and lipids (total cholesterol, TChol; high-density lipoprotein cholesterol, HDL; triglycerides, TG) in serum were measured on Alinity C Immunoassay System (Abbott, IL, USA) using an immunoturbidimetric (kit 07P56 for CRP, Abbott) and enzymatic method (kit 05T88 for TChol, 07P75 for HDL, 07P77 for TG, Abbott, IL, USA). Low-density lipoprotein cholesterol concentrations were calculated by the formula: LDL cholesterol = TChol − [TG/5 + HDL]. Results of laboratory measurements and evaluation of patients were submitted to statistical analysis with PQStat (v.1.6.8.061, PQ-Stat Software). *p*-value below 0.05 was found to be statistically significant.

## 3. Results

Forty patients with skin psoriasis were enrolled into the study. Their characteristics are presented in Table 1. Neither NGAL (*p* = 0.574) nor PCSK9 (*p* = 0.338) showed sex dependance of their levels in serum. Sixteen patients achieved at least 50% reduction of PASI and 24 subjects were classified as non-responders. The treatment caused marked decrease of high-density lipoprotein cholesterol (HDL), primarily in cyclosporine non-responders (Table 2). No statistically significant change was observed for both PCSK9 and NGAL levels in serum before and after the cyclosporine therapy cycle when all patients were analyzed together (Table 3). A significant decrease in the NGAL level in serum after the treatment was visible in the subgroup of patients who responded to cyclosporine therapy. No similar dependance in PCSK9 serum level was observed when responders and non-responders were analyzed separately. The serum NGAL level showed a moderate correlation with BSA and a strong correlation with PASI both at baseline and after treatment (Table 4). Neither PASI nor BSA showed significant correlations with the concentration of PCSK9 in the serum. Only PCSK9 serum level showed moderate to strong correlation with concentrations of total cholesterol (TChol) and LDL at baseline as well as after 3-month of the treatment. Both PCSK9 and NGAL did not exhibit dependance on the CRP level in serum.

## 4. Discussion

Cyclosporine constitutes a standard treatment regimen in psoriasis, but its use may contribute to elevated levels of cholesterol and triglyceride in serum, as well as depletion of HDL, therefore potentiating the risk for cardiovascular disease development [12,13]. The exact mechanisms responsible for cyclosporine-induced hyperlipidemia remain unclear, but cyclosporine was demonstrated to decrease expression of LDL receptors in hepatocytes therefore inhibiting an uptake of LDL [14,15]. Treatment with cyclosporine did not induce changes in the level of LDL in serum, which is regulated by PCSK9. Instead, it caused a decrease in HDL concentrations and thus a development of less favorable lipid profile, which remains in accordance with previous report [13]. Possible increase in the serum level of total cholesterol and LDL was not prevented by the use of 3-hydroxy-3-methylglutaryl coenzyme A reductase inhibitors since their use was exclusion criterium [15].

PCSK9 is primarily known to bind to a transmembrane receptor for LDL on the cell surface leading to its internalization and lysosomal damage in hepatocytes. This enhancement in LDL receptors degradation on hepatocytes promotes accumulation of circulating LDL [3]. This study showed moderate to severe correlation between serum levels of PCSK9 and LDL, as well as TChol. A marked increase of circulating PCSK9 observed previously after acitretin therapy may partially explain pharmacologically induced dyslipidemia in patients receiving the drug [11].

The concentration PCSK9 in serum was previously found to be significantly increased in both human patients with psoriasis and murine model of psoriasis [16]. The expression of PCSK9 was demonstrated to be enhanced in lesioned psoriatic skin where it potentiates proliferation of keratinocytes and their hyperkeratosis, parakeratosis and acanthosis, as well as it prevents keratinocytes from apoptosis. PCSK9 was shown to stimulate inflammatory infiltration and hyperproliferation of epithelial cells in blood vessels of dermis [4]. Circulating PCSK9 showed previously strong correlation with severity of imiquimod model of psoriasis in mice but only moderate correlation with PASI (r = 0.43) in human psoriasis [16]. One should expect its decrease along with the clearance of the skin psoriasis since keratinocytes were identified as the predominant cellular source of PCSK9 in human skin [5], but expectations of the reduction in the serum level of PCSK9 with the improvement of skin psoriasis were not confirmed.

This study showed a significant decrease of serum NGAL concentration among patients responding to cyclosporine therapy. Serum level of NGAL was shown previously to correlate with its tissue expression in lesioned skin in psoriasis [6]. Although NGAL was primarily identified in granules of neutrophils, its expression was previously found to be increased in skin areas associated with disturbed keratinocyte differentiation regardless of the underlying condition [7,17]. It belongs to antimicrobial peptides and expression of NGAL in normal epidermis is low, but a significant enhancement of its expression may occur in different inflammatory skin disorders including psoriasis [1,8]. The molecule may participate in the pathogenesis of psoriasis primarily via the activation of neutrophils [1] and the enhancement of T-helper (Th)1/Th17-mediated inflammation [2]. However, the production of NGAL was found to be augmented by IL-1β, IL-17 and TNF-α [18]. NGAL does not only recruit T cells and neutrophils into skin lesions, but also promotes epidermal parakeratosis [8].

Circulating NGAL was found to be significantly increased in patients with psoriasis and positively correlated with BSA and PASI [19,20]. These observations remain in accordance with our findings on correlations between NGAL and BSA, as well as PASI. Although correlations were found in both clinical evaluation tools, at baseline and after 3-month long cycle of cyclosporine therapy, we are not prone to promote NGAL as a response marker yet. Serum levels of NGAL remained almost the same at the baseline and after the treatment (nb-UVB = 5, anti-TNF alpha = 11) despite the clinical improvement on the skin in the study of Kamata et al. [21]. The change in the serum level of NGAL was not observed by Romani et al. after the nb-UVB with improvement seen on the skin [20]. Median NGAL level in the serum did not change and remained increased after successful topical treatment with 5% salicylic acid ointment and 0.3% anthralin in the study of Baran et al. [22]. These three earlier studies show greater dependance of NGAL production on the activity of systemic inflammation than on the load of keratinocytes or immune cells in the skin. Significantly higher levels of plasma NGAL were previously observed in patients with psoriatic erythroderma and pustular psoriasis than in subjects with chronic plaque subtype [19], but another study did not reveal any correlation between the level of NGAL in serum and indicators of inflammation [22]. Neither NGAL nor PCSK9 showed dependance on the serum CRP level in this study.

The limitation of our research was undoubtedly a low number of enrolled patients, especially since less than half of the group responded to the treatment. The extension of the study group in the future should allow for the analysis of the correlation between PCSK9 or NGAL and psoriasis with difficult-to-treat areas (anogenital, nails, hands and feet, scalp involvement). We cannot exclude that comorbidities in some of our patients could affect laboratory assays of both substances, as well as measurements of lipids. Dietary trends were not analyzed although cholesterol-rich diet was demonstrated previously to potentiate cyclosporine-induced dyslipidemia whereas phytosterols ameliorated it [12,13]. 

There is seen a large diversity of BMI among patients, but only PCSK9 showed earlier correlation with BMI [23]. NGAL showed no significant correlation with BMI in both patients with psoriasis [21,22,24] and subjects with metabolic syndrome [25]. Neither serum level of PCSK9 (r = 0.03; *p* < 0.87) nor of NGAL (r = 0.02; *p* = 0.91) showed correlations with BMI among our patients. One should notice that psoriasis predisposes to metabolic syndrome and there was seen increased BMI (pre-obesity) both in men and women (Table 1). This might indicate a better choice of other than cyclosporine treatment options for our patients. Biological drugs targeting IL-23/Th-17 immune signaling pathway probably helps to limit the susceptibility to development of metabolic and cardiovascular diseases in patients with psoriasis [26], but their use typically needs to be preceded by standard treatment regimens (cyclosporine, methotrexate, acitretin or PUVA). Apremilast was shown to affect metabolic inflammation and insulin resistance contributing to reduction of glucose level in those psoriatic patients with hyperglycemia [27]. DMF was demonstrated to exert antioxidative activity in psoriasis whereas oxidative stress is known to promote adipose tissue deposition and development of obesity [28]. Both these small molecule drugs are not routinely available in Poland. 

A treatment with acitretin preceding cyclosporine therapy reported in four subjects could influence somehow on their lipid status and laboratory measurements due to a long wash-out period. The exclusion of these patients did not cause a loss or a gain of statistical significance for the majority of analyzed parameters, except for LDL. The concentration of LDL in serum showed a strong tendency to be significantly reduced after 3-months of treatment with cyclosporine, but not when responders and non-responders were analyzed separately (Supplementary File). Finally, the use of topical ointments was not forbidden at the enrollment to the study, but these factors could influence primarily the response efficacy of cyclosporine therapy as well as laboratory assays. However, median NGAL level did not change after successful topical treatment with 5% salicylic acid ointment and 0.3% anthralin in the study of Baran et al. [22].

## 5. Conclusions

The cyclosporine therapy contributes to the reduction in the level of NGAL in serum but not the one of PCSK9. Serum level of NGAL shows moderate to strong correlation with BSA and PASI, respectively, whereas PCSK9 remains independent on the severity of psoriasis, but it correlates with the levels of TChol and LDL in serum. The last finding may help to understand a drug induced dyslipidemia after some systemic treatment options in psoriasis.

## Figures and Tables

**Table 1:** Clinical characteristics of patients.

	Women	Men	*p*-Value
number	17	23	
age median (range) [years]	48 (27–58)	41 (31–59)	0.42 *
BMI median (range) [kg/m^2^]	27.6 (19.1–39.3)	27.7 (19.4–38.3)	0.8 *
arterial hypertension	5 (29%)	4 (17%)	0.46 Fisher **
cardiovascular comorbidities	2 (12%)	3 (13%)	1.0 Fisher **
diabetes mellitus	2 (12%)	4 (17%)	1.0 Fisher **
cardiac treatment	5 (29%)	5 (22%)	0.72 Fisher **
elevated CRP	5 (29%)	3 (13%)	0.25 Fisher **
PASI median (range)	19.6 (13.4–38.2)	17.8 (8.7–36.8)	0.65 *
BSA median (range)	36 (21–90%)	34 (14–74)	0.57 *
lipids [mmol/L] -TChol -HDL -LDL -TG	5.3 ± 0.9 1.3 ± 0.3 3.9 ± 1.1 1.7 ± 0.6	5.3 ± 1.2 1.3 ± 0.3 4.0 ± 1.2 1.8 ± 0.9	0.99 *** 0.49 *** 0.88 *** 0.65 ***
previous treatment other than topical -acitretin -methotrexate -phototherapy -none	1 (18%) 5 (24%) 8 (29%) 9 (29%)	3 (4%) 4 (22%) 5 (35%) 5 (39%)	0.65 **

* U Mann–Whitney test; ** Fisher exact test; *** t-Student test.

**Table 2:** Change of psoriatic characteristics and lipids levels before and after treatment.

	PASI	BSA	TChol	HDL	LDL	TG
**all patients together** (n = 40)						
before	18.4	35.0	5.2 ± 1.1	1.3 ± 0.3	4.0 ± 1.1	1.8 ± 0.8
after	14.2	22.5	5.1 ± 0.9	1.2 ± 0.2	3.6 ± 0.9	1.9 ± 0.7
*p*-value	<0.001	<0.001	0.58	0.02	0.1	0.4
**responders to cyclosporine** (n = 16)						
before	21.3 ± 7.6	43.0 ±17.0	5.35 ± 1.0	1.2 ± 0.2	4.2 ± 1.0	1.85 ± 0.6
after	4.2 ± 3.0	6.0 ± 3.5	5.14 ± 0.8	1.1 ± 0.2	3.6 ± 0.8	2.0 ± 0.5
*p*-value	<0.001	<0.001	0.51	0.41	0.1	0.53
Δ [%] (range)	80 (61–99)	84 (68–97%)				
**non-responders to cyclosporine** (n = 24)						
before	18.0	34.0	5.2 ± 1.1	1.3 ± 0.3	3.8 ± 1.2	1.7 ± 0.8
after	16.3	26.0	5.1 ± 1.0	1.2 ± 0.2	3.6 ± 1.0	1.8 ± 0.8
*p*-value	0.29	0.03	0.83	0.02	0.41	0.55
Δ [%] (range)	11 (−22–22)	17 (−17–33)				

U Mann–Whitney test or t-Student test.

**Table 3:** Serum level of PCKS9 and NGAL before and after cyclosporine treatment.

	PCSK9 [ng/mL]			NGAL [pg/mL]		
	Before	After	*p*-Value	Before	After	*p*-Value
all patients	66	67	0.81	2469	2330	0.65
non-responders	61	69	0.17	2291	3441	0.09
responders	83	65	0.15	3277	1581	0.0066

U Mann–Whitney test.

**Table 4:** Correlations between psoriasis characteristics, lipid levels, PCSK9, and NGAL at baseline and after cyclosporine treatment.

	PCSK9 r (*p*-Value)		NGAL r (*p*-Value)	
	Before	After	Before	After
PASI	0.004 (0.97)	0.13 (0.44)	0.87 (<0.001)	0.7 (<0.001)
BSA	−0.04 (0.79)	0.19 (0.25)	0.47 (0.002)	0.52 (<0.001)
TChol	0.65 (<0.001)	0.42 (0.006)	0.08 (0.62)	−0.14 (0.4)
HDL	−0.04 (0.79)	−0.11 (0.51)	0.07 (0.66)	0.29 (0.06)
LDL	0.61 (<0.001)	0.44 (0.004)	0.06 (0.7)	−0.15 (0.36)
TG	0.08 (0.47)	−0.12 (0.48)	0.08 (0.6)	0.0 (0.98)

Spearman’s correlation coefficient.

## Data Availability

On the demand to corresponding author after e-mail contact.

## Supplementary Materials

The following supporting information can be downloaded at: https://www.mdpi.com/article/10.3390/life13091873/s1.
