# Supplementary material for: Significance of Neutrophil Gelatinase-Associated Lipocalin (NGAL) and Proprotein Convertase Subtilisin/Kexin Type 9 (PCSK9) for the Monitoring of Treatment Response to Cyclosporine in Patients with Psoriasis
Source: Life (Basel). 2023 Sep 6;13(9):1873. doi: 10.3390/life13091873 (PMC10532527; doi:10.3390/life13091873)
Supplement: Supplementary file 1 [file life-13-01873-s001.zip › life-2579531-supplementary.pdf]

life

MDPI

Type of the Paper (original research manuscript.)

Significance of neutrophil gelatinase-associated lipocalin (NGAL) and proprotein convertase subtilisin/kexin type 9 (PCSK9) for the monitoring of treatment response to cyclosporine in patients with psoriasis.

- supplementary file -

Change of psoriatic characteristics and lipids levels before and after the treatment when patients with previous acitretin therapy were excluded.

	PASI	BSA	TChol	HDL	LDL	TG
all patients together (n=40)						
before	18.4	35.0	5.4 ± 1.0	1.3 ± 0.2	4.1 ± 1.1	1.8 ± 0.8
after	14.4	22.5	5.2 ± 0.9	1.1 ± 0.2	3.7 ± 0.9	1.9 ± 0.7
p-value	<0.001	<0.001	0.52	0.03	0.08	0.5
responders to cyclosporine (n=14)						
before	22.0 ± 7.6	44.0 ± 18.1	5.6	1.2	4.3	1.8
after	4.6 ± 3.0	6.0 ± 3.5	5.4	1.1	3.7	1.85
p-value	<0.001	<0.001	0.7	0.37	0.1	0.5
Δ [%] (range)	79 (61-99)	81 (68-97%)				
non-responders to cyclosporine (n=24)						
before	19.6 ± 6.5	34.0	5.1	1.3 ± 0.3	4.0 ± 1.1	1.7 ± 0.9
after	18.0 ± 5.4	26.0	5.0	1.2 ± 0.2	3.7 ± 1.0	1.8 ± 0.8
p-value	0.38	0.02	0.63	0.04	0.3	0.6
Δ [%] (range)	14 (-3-22)	19 (-14-33)				

U Mann-Whitney test or t-Student test

Serum level of PCKS9 and NGAL before and after the treatment when patients with previous acitretin therapy were excluded.

	PCSK9 [ng/mL]			NGAL [pg/mL]		
	before	after	p-value	before	after	p-value
all patients	70	67	0.89	2677	2498	0.74
non-responders	60	65	0.23	2292	3850	0.07
responders	89	69	0.14	3530	1782	0.006

U Mann-Whitney test

Correlations between characteristics of psoriasis, lipids levels and PCSK9, NGAL, at the baseline and after the treatment with cyclosporine, when patients with previous acitretin therapy were excluded.

	PCSK9 r (p-value)		NGAL r (p-value)	
	before	after	before	after
PASI	0.004 (0.99)	0.02 (0.89)	0.86 (<0.001)	0.68 (<0.001)
BSA	-0.04 (0.82)	0.08 (0.65)	0.42 (0.01)	0.51 (<0.001)
TChol	0.67 (<0.001)	0.46 (0.004)	- 0.04 (0.83)	-0.33 (0.05)
HDL	-0.04 (0.79)	-0.14 (0.41)	0.19 (0.27)	0.38 (0.02)
LDL	0.65 (<0.001)	0.5 (0.002)	- 0.05 (0.78)	-0.33 (0.04)
TG	0.07 (0.68)	-0.17 (0.31)	0.01 (0.93)	-0.06 (0.7)

Spearman's correlation coefficient
